# Breakage of fascial closure device during laparoscopic surgery

**DOI:** 10.4103/0972-9941.16533

**Published:** 2005-06

**Authors:** A. N. Katara, D. S. Bhandarkar, R. S. Shah, T. E. Udwadia

**Affiliations:** Department of Minimal Access Surgery, P. D. Hinduja National Hospital & Medical Research Centre, Veer Savarkar Marg, Mahim, Mumbai 400016, India

**Keywords:** Laparoscopic surgery, instrument, breakage

## Abstract

Breakage of instruments during laparoscopic surgery is rare. However, when it does occur, locating and retrieving the broken part of the instrument can be cumbersome. Moreover, inability to do so may carry serious medicolegal implications. We report a patient in whom the tip of a fascial closure device broke during laparoscopic surgery. This was located by intraoperative fluoroscopy and retrieved from the extraperitoneal plane via a small incision. The paper discusses the probable factors responsible for breakage of the fascial closure device in our patient and reviews the previously reported cases of the rare complication of breakage of instruments during laparoscopic surgery.

## INTRODUCTION

Breakage of instruments during laparoscopic surgery is rare. However, when it does occur retrieval of the broken part can test the ingenuity of the surgeon. Inability to retrieve a broken part may carry serious medicolegal implications. We report a patient in whom the tip of a fascial closure device (FCD) broke intraoperatively. The likely factors responsible for this adverse event are discussed and the literature reviewed to identify cases of instrument breakage occurring during laparoscopic surgery.

## CASE REPORT

A 43-year-old lady was scheduled to undergo laparoscopic biopsy of a lymph nodal mass situated along the superior border of the pancreas suspected to be tuberculous in origin. During laparoscopy, the nodal mass was visualized through the gastrohepatic omentum. A falciform lift[1] was considered necessary to elevate the bulky falciform ligament hanging down onto the operative field. A suture held in an indigenously manufactured FCD was introduced to the left of the falciform through a stab incision. As the FCD carrying a polyglycolic acid suture was pushed through the abdominal wall and a sharp spike was seen entering the abdominal cavity, it became apparent that the jaw mechanism at the tip had broken and had lodged itself along the track. The lymph node biopsy was completed without falciform lift and the specimen was retrieved. At this stage, intraoperative fluoroscopy was utilized to identify the position of the tip, which was found lying just under the entry point of the FCD. A 3-cm skin incision was made, deepened through the subcutaneous tissue and the rectus sheath. The broken tip lodged in the extraperitoneal fat was identified with the help of a haemostat used for probing the wound. This was grasped and retrieved. The fascia was closed with a non-absorbable suture material and all skin incisions were sutured. The patient made an uneventful recovery and was discharged 36 hours later. The event was discussed with the patient and her family postoperatively and documented in the case records. She remains well at follow up six months later.

## DISCUSSION

Intraoperative breakage of instruments is rare during open surgery, probably because of the robust construction of open surgical instruments which lack multiple, movable parts. If an instrument does break during open surgery it is more likely to be noticed promptly and retrieved. Laparoscopic instruments, including the reusable “take-apart” metal variety, have more components as compared to their open counterparts and are more delicate. Be as that may, instrument breakage is uncommon during laparoscopic surgery.

**Figure 1:**
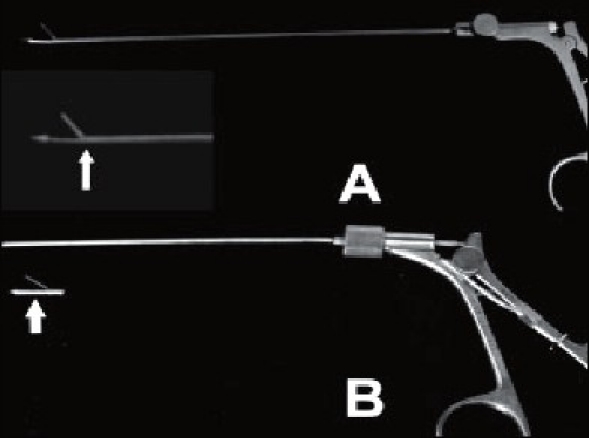
(A) Lower jaw of the Berci fascial closure device is continuous with the shaft (arrow in inset). (B) Broken tip (arrow) comprising upper and lower jaws soldered on to the body of the indigenous fascial closure device

Laparoscopic FCD / suture passer is a thin instrument with a sharp tip. We have regularly used this device for closure of fascia at the site of ports as also for exteriorizing stay sutures without any complication in over 2000 cases. After the adverse incident reported above, we carefully inspected the tips of the Berci FCD (Karl Storz, Tuttlingen, Germany) (Figure 1A) and also of the indigenous device manufactured locally that was used in our patient (Figure 1B). In the Berci device, the lower jaw of the tip is an extension of the body of the instrument, whereas the upper movable jaw functions on a hinge. In the device locally manufactured device we had used, the tip consisting of two jaws was detachable and appeared to have been soldered on to the shaft. The FCD, both the Berci and indigenous variety, have a groove in which the suture must be placed. If the suture is not placed in the groove, the profile of the tip is not a smooth tapering point. Pushing such a tip through tissues may require use of excessive force to be applied leading to breakage of the tip. It is unclear whether in our case improper positioning of suture in the groove of the FCD was a factor responsible for breakage of its tip.

Almost all laparoscopic instruments are introduced into the abdominal cavity through ports and thus their tips do not meet with any degree of resistance. In our patient, introduction of the FCD percutaneously through a stab incision may have resulted in generation of excessive force that caused it to break at its weakest point - where the tip was soldered on to the shaft. During arthroscopic procedures, thin instruments with delicate, sharp tips are introduced percutaneously to manipulate intra-articular structures, and breakage of the tip of these instruments is a well-recognised complication.[2] To guard against this eventuality in laparoscopic surgery, it is imperative to check that delicate instruments such as a FCD do not have soldered parts at the tip which make them prone to breakage. Furthermore, if during manufacture or repair of a laparoscopic instrument the inexpensive lead-based or silver solder is used, it weakens the instrument because it is applied at high temperatures. After the use of such solder, the instrument is more likely to break.[3] Repeated autoclaving of reusable instruments may weaken them and make them prone to breakage. Routine and thorough checking of the instruments by those responsible for their maintenance can help avoid intraoperative breakage.

Recently, Salameh reported two cases of breakage of the tip of Gore suture passer (W. L. Gore, & Associates, Newark, DE) during laparoscopic repair of ventral hernia.[4] This author points out that a common mechanism of breakage of suture passer is its withdrawal in a partially open position such that the tip catches on the fascia and breaks. Also, changing the direction of the suture passer half way through its introduction is liable to generate opposing shear forces on the tip resulting in its breakage. Thus, both these points need to be guarded against when using a suture passer to avoid breakage of its tip.

If an instrument breaks during laparoscopic surgery and the broken part lies in the operative field, it can be retrieved immediately. For example, Lynch et al reported the breakage and recovery of a 2-mm segment of needle from an Autosuture Endostitch device (U.S. Surgical) during a laparoscopic Burch procedure which was retrieved uneventfully.[5] Often, however, the breakage becomes apparent after some time or the broken part migrates in the abdominal cavity, making its retrieval difficult. Use of intraoperative fluoroscopy, as was done in our case, is an option to locate and remove the broken instrument part. Kandioler-Eckersberger et al describe the use of a novel method to identify the tip of a reusable laparoscopic grasper which was lost between loops of bowel in two cases.[6] After initial unsuccessful attempts at retrieval by patient positioning, fluoroscopic localization, endoscopic visualization etc, they used a magnetic probe to retrieve the broken parts. The probe consisted of a 6-cm magnetic tip attached to a 40-cm semi flexible Teflon rod. The probe was passed through one of the ports and was placed in the vicinity of the lost part under fluoroscopic guidance; the lost metallic piece was attracted to the magnet. It was retrieved and a laparotomy was avoided.

The action that needs to be taken after an adverse event involving breakage of a medical device varies from hospital to hospital. It is essential to document the event in the operation records and report it to the hospital authority. In the Western countries it may also have to be reported to the relevant state authority in prescribed forms.[7] Discussion with the patient of the complication and the remedial measures taken helps put the event in perspective and may reduce the likelihood of a legal action. If appropriate, the information should be passed on to the insurance company indemnifying the surgeon and their opinion sought at an early stage.

In conclusion, breakage of instruments is rare during laparoscopic surgery but may occur with thin, sharp instruments introduced percutaneously. Periodic inspection of delicate instruments and their careful maintenance and use should help guard against such mishaps.
